# Effects of using a cognitive aid on content and feasibility of debriefings of simulated emergencies

**DOI:** 10.3205/zma001491

**Published:** 2021-06-15

**Authors:** Julia Freytag, Fabian Stroben, Wolf E. Hautz, Dorothea Penders, Juliane E. Kämmer

**Affiliations:** 1 Charité - Universitätsmedizin Berlin, corporate member of Freie Universität Berlin and Humboldt-Universität zu Berlin, Simulated Patient Programme, Berlin, Germany; 2 Charité - Universitätsmedizin Berlin, corporate member of Freie Universität Berlin and Humboldt-Universität zu Berlin, Department of Anesthesiology and Operative Intensive Care Medicine (CBF), Berlin, Germany; 3 Inselspital University Hospital Bern, Department of Emergency Medicine, Bern, Switzerland; 4 Charité - Universitätsmedizin Berlin, corporate member of Freie Universität Berlin and Humboldt-Universität zu Berlin, Lernzentrum, Berlin, Germany; 5 Charité - Universitätsmedizin Berlin, corporate member of Freie Universität Berlin and Humboldt-Universität zu Berlin, Department of Anesthesiology and Operative Intensive Care Medicine (CCM, CVK), Berlin, Germany

**Keywords:** teamwork, non-technical skills, structured debriefing, cognitive aid, simulation-based education

## Abstract

**Background: **Adverse events in patient care are often caused by failures in teamwork. Simulation training and its debriefing can contribute to improving teamwork and thus patient care. When conducting debriefings, there are several design factors that can potentially influence learning outcomes. This study examines the use of a cognitive aid to help structure the content of debriefings and compares it with debriefings that are merely roughly structured. In addition, the feasibility of the debriefing, the satisfaction of the participants and their teamwork during the training are investigated.

**Methods: **In a simulated night shift, seven teams of four to five medical students (n=32) took part in six cases that simulated common situations in an emergency medicine environment and received a debriefing on their teamwork after each case, either in the intervention condition with the help of the TeamTAG tool – a cognitive aid focusing on selected teamwork principles from Crisis Resource Management (CRM) – or in the control condition without it. The facilitators noted the topics of the debriefings and rated their experience of conducting them; the participants indicated their satisfaction with the debriefings, as well as their assessment of the importance of CRM principles. In addition, the quality of teamwork was assessed using the Team Emergency Assessment Measure (TEAM).

**Results: **The analysis showed no difference in the number of teamwork principles discussed between the control and intervention conditions, but topics were repeated more frequently in the control group. The TeamTAG guideline was focused on and implemented by the tutors, who discussed the CRM principles included in the TeamTAG more consistently than in the control condition. The tutors in both conditions were satisfied with the implementation, and the use of TeamTAG facilitated time management. There were no differences in participants’ satisfaction, their assessment of the importance of the teamwork principles, or the quality of teamwork between conditions.

**Conclusion:** The use of a cognitive aid can help to direct the focus on certain topics or learning objectives and facilitate time management through pre-structuring; however, a difference in learning outcomes (in terms of the quality of teamwork) could not be identified. Besides the influence of a certain structure or script, a strong influence from the individual guiding the debriefing is likely.

## Zusammenfassung

**Zielsetzung: **Unerwünschten Ereignissen in der Patientenversorgung liegen häufig Versäumnisse in der Teamarbeit zugrunde. Simulationstrainings und deren Nachbesprechung, das Debriefing, können einen Beitrag zur Verbesserung der Teamarbeit und somit der Versorgung leisten. Bei der Durchführung von Debriefings gibt es verschiedene Gestaltungsfaktoren, die den Lernerfolg möglicherweise beeinflussen. In dieser Studie wird der Einsatz eines strukturierenden Leitfadens auf den Inhalt von Debriefings untersucht und mit nur grob strukturierten Debriefings verglichen. Darüber hinaus wird die Durchführbarkeit des Debriefings, die Zufriedenheit der Teilnehmenden und ihre Teamarbeit im Verlauf des Trainings untersucht.

**Methodik: **In einem simulierten Nachtdienst durchliefen sieben Teams von vier bis fünf Medizinstudierenden (n=32) insgesamt sechs Fälle aus der Notfallmedizin und erhielten nach jedem Fall ein Debriefing zu ihrer Teamarbeit: entweder in der Interventionsbedingung unter Zuhilfenahme des Leitfadens TeamTAG, der ausgewählte Teamarbeitsprinzipien des Crisis Resource Management (CRM) in den Fokus stellt, oder in der Kontrollbedingung ohne diesen. Die debriefenden Tutor*innen notierten die Themen der Debriefings und bewerteten ihre Erfahrungen mit der Durchführung; die Teilnehmenden gaben ihre Zufriedenheit mit dem Debriefings, sowie ihre Einschätzung zur Wichtigkeit der CRM-Prinzipien an. Zusätzlich wurde die Qualität der Teamarbeit mit dem Team Emergency Assessment Measure (TEAM) erfasst.

**Ergebnisse: **Zwischen Kontroll- und Interventionsbedingung gab es keinen Unterschied in der Anzahl der besprochenen Teamarbeitsprinzipien, jedoch kam es in der Kontrollgruppe häufiger zur Wiederholung von Themen. Der durch den TeamTAG gesetzte inhaltliche Schwerpunkt wurde von den Tutor*innen umgesetzt, sie besprachen die dort enthaltenen CRM-Prinzipien konsistenter als in der Kontrollbedingung. Die Tutor*innen beider Bedingungen waren mit der Durchführung zufrieden, die Nutzung des TeamTAG erleichterte dabei das Zeitmanagement. Es gab keine Unterschiede bezüglich der Zufriedenheit der Teilnehmenden, ihrer Einschätzung der Wichtigkeit der Teamarbeitsprinzipien oder der Qualität der Teamarbeit zwischen den Bedingungen.

**Schlussfolgerung: **Die Nutzung eines Debriefing-Leitfadens kann helfen, den Fokus auf bestimmte Themen oder Lernziele zu lenken und durch die Vorstrukturierung das Zeitmanagement erleichtern; ein Unterschied im Lernerfolg (i.S. der Qualität der Teamarbeit) zeigt sich hingegen nicht. Neben dem Einfluss einer bestimmten Struktur oder eines Leitfadens ist ein starker individueller Einfluss der debriefenden Person wahrscheinlich.

## 1. Introduction

Errors in the diagnosis and treatment of patients occur regularly and can cause harm or even death [1], [2]. The proportion of hospital patients who experience so-called adverse events is 5-10% – about half of which are classed as preventable [3]. The causes of preventable adverse events often lie in the area of non-technical skills (NTS) [4], [5], [6]. These skills include communication, leadership, collaboration, and decision making – often subsumed under the term teamwork – and describe all interactions that occur between team members when jointly addressing a task. Together with the individual work of each team member, also called taskwork, in which technical skills and knowledge are brought to bear, this results in the team’s performance [7], [8]. Several studies have shown that good teamwork reduces the risk of adverse events occurring [7], [9].

Simulation-based trainings (SBTs) have been shown to be a suitable format to improve team performance by training technical and non-technical skills [10], [11]. They provide the opportunity for learners to practise their skills in a safe environment [12]. In addition to skills training, simulation training aims at imparting or consolidating knowledge and it also considers participants’ attitude towards a topic (often described as the triad “knowledge, skills and attitude”, or KSA) [13]. Numerous studies have confirmed the positive effects of SBTs and indicate improved clinical outcomes such as lower complication rates and morbidity [10], [14], [15].

A key component of simulation training is debriefing, which is a process of (guided) reflection on a simulated scenario [16]. This reflection represents an important part of the learning process in terms of experience-based learning [17]. Feedback, which compares learners’ performance against a known standard, is often a crucial component of debriefing and has been shown to be notably effective [11], [18], [19].

There are different types of debriefing, depending on their goals, timing and structure [19], [20]. Very common are debriefings that take place directly after a simulation has ended. Often, three-phase models are used, in which the participants first report their experiences and collect observations. Then certain sequences are analysed in more detail and errors, their causes and ways how to avoid them in the future are discussed. Finally, the discussion is summarised and goals are set for further collaboration as a team. This structure is known as GAS (gather – analyse – summarise) [21], [22]. In addition, so-called cognitive aids are used to support the debriefing process. These are scripts or guidelines that list the most important steps during specific (emergency) situations. In practice, they commonly take the form of posters, for example, and have proven to be helpful in many clinical areas [23], [24], [25].

It is uncertain which design factors relating to debriefings have an influence on learning success [16], [26]. For example, it is unclear whether a stronger (content-related) pre-structuring with the help of a cognitive aid can increase learning success compared to a free or merely roughly structured debriefing (that is, on the basis of the GAS guideline but without the cognitive aid described below). An important aspect is the possible effect of pre-structuring on the content and scope of the topics discussed in a debriefing. Initial studies suggest that using a cognitive aid during debriefings has a positive effect on learning success, especially when applied by inexperienced facilitators [27], because these types of aid can provide practical and specific suggestions as to which aspects can be observed and debriefed on. The present study serves to further clarify the influence of a debriefing supported by a debriefing guideline on the feasibility of the procedure, examines the satisfaction and attitude of the participants, and investigates their teamwork [28].

As a cognitive aid, a guideline called “**TEAM**work **T**echniques **A**nalysis **G**rid (TeamTAG)” was developed by the working group [28]. TeamTAG uses behavioural anchors to support observations and provides a structure for debriefing. This is to ensure that all learning objectives are discussed during a simulation training and to support the debriefing process from the facilitator’s perspective. The guideline lists six Crisis Resource Management (CRM) principles [29] that aim to help teams respond to crises [30]. Knowing these principles is an elementary first step towards their conscious application – many trainings in continuing education are based on CRM principles [14] and show positive effects in terms of mastering these principles, such as enhanced team communication and management [31].

To compare the pre-structured debriefing using a guideline and the merely roughly structured debriefing according to GAS, we formulated the following research questions:

1. What influence does the use of a guideline have on the scope and content of debriefings on teamwork?
2. How do participants and facilitators evaluate debriefings with a guideline compared to merely roughly structured debriefings?
3. What influence does the use of a debriefing guideline have on participants’ attitudes towards the CRM principles?
4. Can the use of a debriefing guideline lead to an immediate improvement in teamwork?

## 2. Methods

### 2.1. Study design

The study was planned as a randomised controlled trial and conducted at the local skills lab at Charité Universitätsmedizin Berlin. The research design has already been published as a study protocol [28].

**Figure 1:**
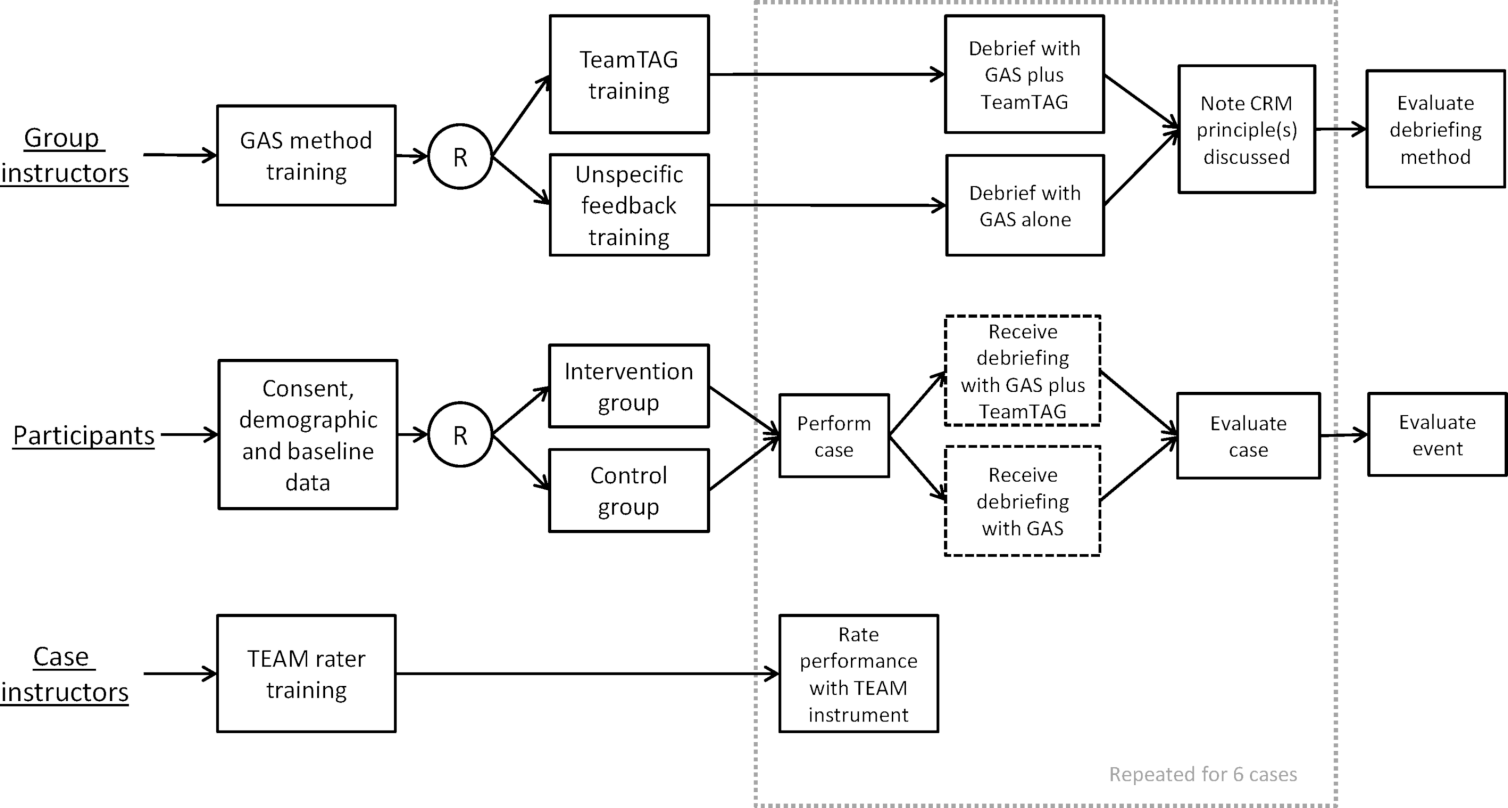
Study design (R = randomization. This figure was published as part of the referenced study protocol [28], and is used under CC BY-NC 4.0; it has been adapted for use in this publication.)

During a simulated night shift in an emergency department, teams of four to five students worked through six simulation scenarios. The simulation started in the evening and continued throughout the night to make it as realistic as possible and to give students the opportunity to experience the stress of working at night. Directly after each scenario, they received a debriefing on their teamwork (see figure 1 (Fig. 1)) [32]. Teams were randomised to one of two conditions: in the intervention condition, the debriefing took place according to the GAS structure and using the debriefing TeamTAG guideline. In the control condition, debriefing was conducted according to GAS but without further guidance. 

#### 2.2. TeamTAG

TeamTAG lists six CRM principles, each with associated observable behaviours. The principles were selected to match the simulation scenario, the participants’ skill level, the facilitators’ level of experience, and the observability of the principles during the simulation.

TeamTAG can be found in attachment 1 ; a feasibility study was published as part of the study protocol [28].

#### 2.3. Participants

Medical students who had completed the tenth semester of their studies in human medicine (before or after the second part of the state examination) and who had voluntarily registered for the study were included. All participants were informed in writing and verbally about the study’s objective and procedure before it was carried out. The study was approved by a Data Protection Officer (AZ 737/16) and the Charité Ethics Committee (EA2/172/16). According to the calculation of power and sample size, six teams would be necessary to detect a meaningful difference between conditions, defined as a significant improvement of 11 points in the TEAM sum score (one point per item, see 2.6.4) [28].

#### 2.4. Simulation scenarios

All teams rotated through six simulation scenarios. These represented common and important cases in emergency medicine where teamwork is highly relevant (such as resuscitation). Due to the rotation principle, the order of the cases varied depending on the team [32]. Before the start of each case, the students designated a team leader and two team members to handle the case; the remaining one or two team members observed the simulation. These changes in team structure allowed the students to experience different roles and their associated challenges over the course of the simulated night shift.

#### 2.5. Debriefing of teamwork

Each case was followed by a ten-minute debriefing of the teamwork by one facilitator per team according to the GAS principle [21], [22]; in each debriefing, one or more CRM principles were discussed in the analysis phase.

The seven facilitators were advanced medical and nursing students who were also experienced peer tutors. In preparation, all tutors received training in the CRM concept and on debriefing according to the GAS structure. The intervention group facilitators also received an introduction to the use of TeamTAG and were instructed to address all six CRM principles of TeamTAG during the first five debriefings. The control group facilitators were instructed to choose any of the CRM principles as the topic of the debriefing – matched to what they observed in the simulation. Furthermore, facilitators in both conditions were instructed to repeat any content if necessary.

#### 2.6. Measures

##### 2.6.1. Initial survey

Participants completed an initial survey on prior emergency medicine experience and on demographic variables. Participants then discussed in their teams what principles of teamwork they already knew (CRM baseline). Afterwards, each team collectively solved 15 multiple-choice questions on emergency medicine to assess prior medical knowledge.

##### 2.6.2. Content and scope of debriefings

After each debriefing, the instructors noted the topics they discussed with the participants.

##### 2.6.3. Satisfaction with debriefings

After each debriefing, participants rated whether they found it helpful (ranging from +3: strongly agree to -3: strongly disagree).

##### 2.6.4. Quality of teamwork

The Teamwork Emergency Assessment Measure (TEAM) was used to evaluate teamwork [33]. It was developed for use in simulated and ‘real’ emergency scenarios [34], [35] and the original English version and the French translation demonstrate excellent psychometric properties [36]. In an earlier study by the working group, TEAM was translated into German and validated [37].

TEAM evaluates the behaviour of the whole team by means of 11 items in three categories on 5-point Likert scales (0: behaviour was never/almost never shown, 4: behaviour was always/almost always shown). The assigned ratings can be added up to a sum score (0-44). In addition, overall performance was assessed by means of a global rating scale or GRS (1: very poor performance, 10: very good performance). During the study, teamwork was evaluated in every scenario by one student rater and one professional rater (a physician or psychologist with experience in emergency medicine and in medical simulations), and the mean of both ratings was used for the analyses. A total of six student raters and six professional raters, who had previously been trained in the use of TEAM, participated.

##### 2.6.5. Final survey

After completing all simulations, participants rated how relevant they thought the 15 CRM principles were (+3: very relevant, -3: not relevant at all).

#### 2.7. Analysis

The demographic characteristics of both the intervention and the control conditions were compared to identify potential confounders. The participants’ prior knowledge of teamwork and the information provided by the tutors on the CRM principles they had addressed during debriefings were mapped to the 15 CRM principles independently by two raters and then agreed upon. 

Possible differences between the conditions were examined using Chi-square tests, t-tests, and an analysis of covariance (ANCOVA). 

## 3. Results

### 3.1. Demographic data/confounder

Thirty-two medical students participated in the study (see table 1 (Tab. 1)). They were randomised into four intervention groups (n=19; three groups of 5 participants each, one of 4 participants) and three control groups (n=13; one group of 5 participants each, two of 4 participants).

The participants under the intervention and control conditions did not differ in their demographic characteristics, prior experience, or emergency medicine knowledge level (see table 1 (Tab. 1)). However, teams in the control condition knew more CRM principles at baseline. However, this was not associated with better teamwork in the first case.

#### 3.2. Scope of debriefings

**Figure 2:**
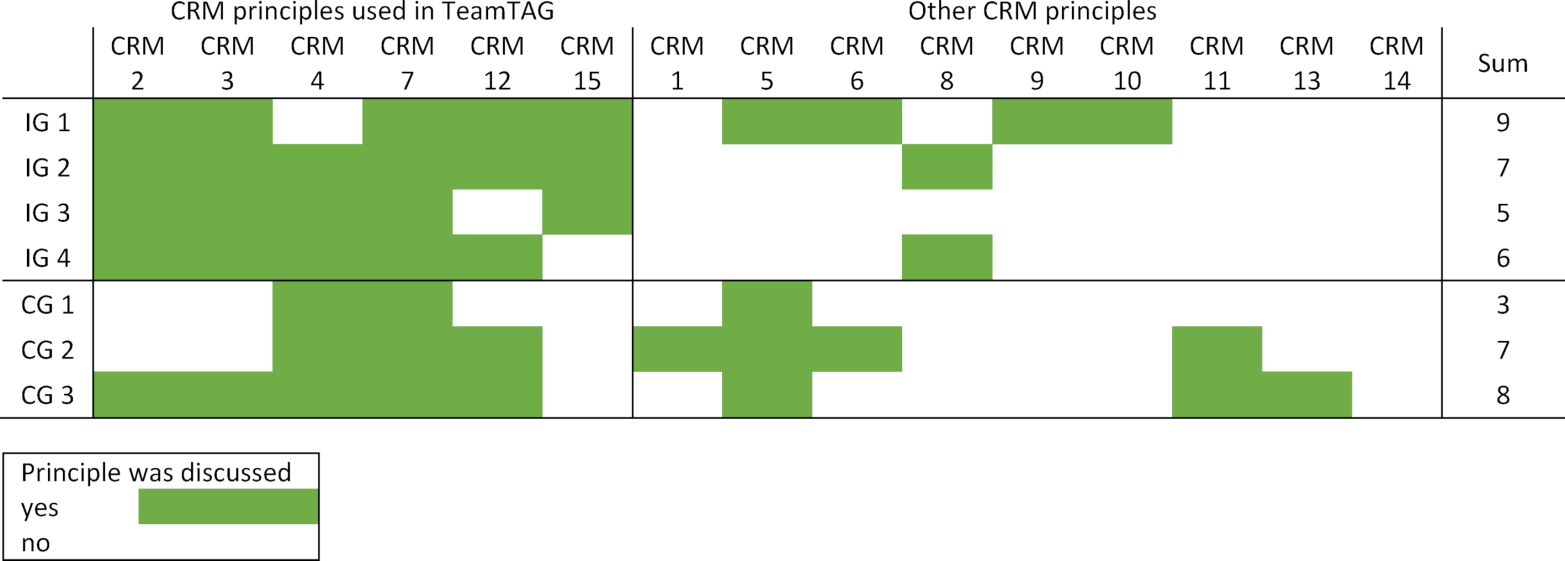
CRM principles discussed by intervention and control groups, considering the TeamTAG focus (IG = intervention group, CG = control group. The numbering of the CRM principles is based on the list of 15 CRM principles according to Rall and Gaba [29], [43].)

In total, the groups in the intervention condition discussed an average of M=7.50 principles (SD=1.29) and M=6.33 principles (SD=3.06) in the control condition,* t*(5)=- 0.70, p=.51 (see figure 2 (Fig. 2)).

#### 3.3. Content of debriefings

As can be seen in figure 2 (Fig. 2), the CRM principles listed in the TeamTAG were discussed more consistently in the intervention condition than in the control condition; additional principles, on the other hand, were rarely talked about. In the intervention condition, the groups discussed a median of 5 (min=4; max=6) of the six TeamTAG principles; in the control condition, a median of 3 (min=2; max=5) principles.

**Figure 3:**
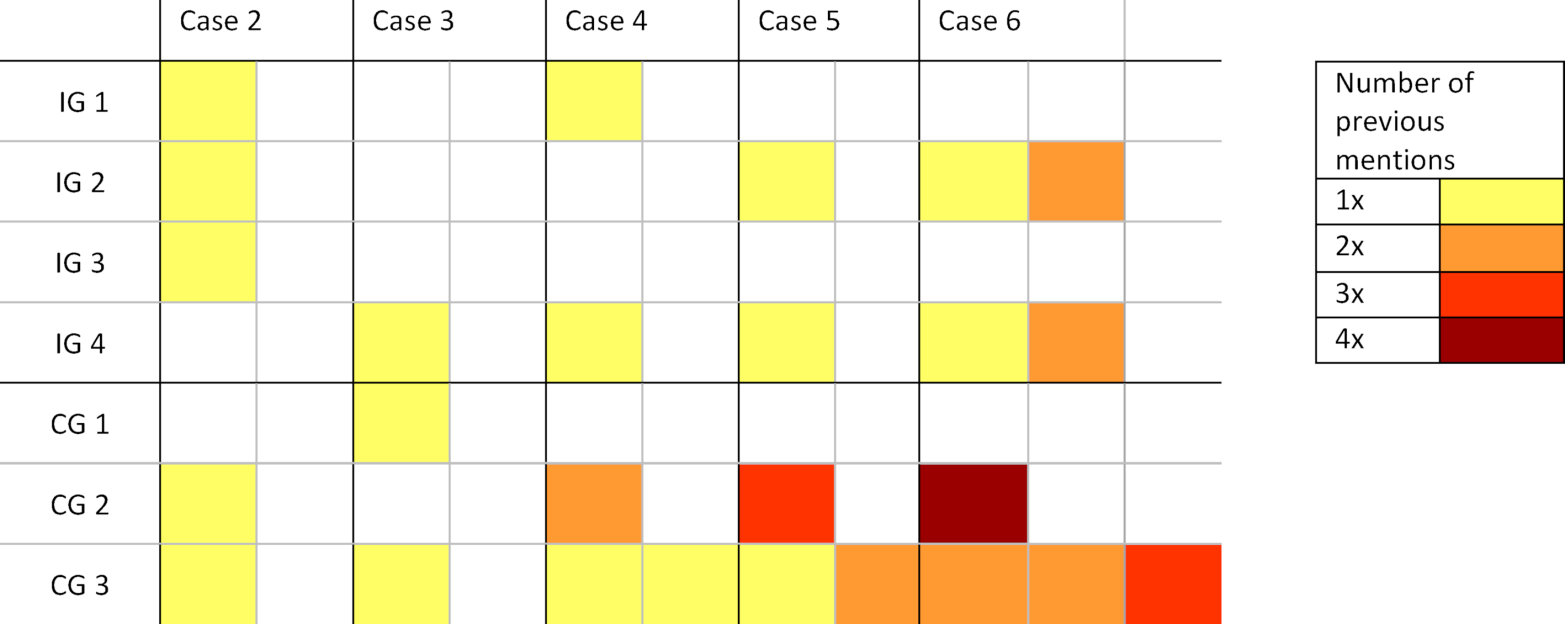
Heatmap showing the repetition pattern of feedback topics in each group (IG = intervention group, CG = control group)

Figure 3 (Fig. 3) breaks down how often principles were repeated. It shows that, within the intervention group, ‘new’ topics were mostly discussed in each debriefing. An exception is case 6, where a repetition was instructed. The control groups, on the other hand, more frequently repeated CRM principles, especially the principles 4, 7 and 12 (role of leadership or team member, safe communication and re-evaluating situations), which are also mentioned in TeamTAG. 

#### 3.4. Facilitators’ rating of TeamTAG

Facilitators in both conditions stated that they were able to observe and debrief the teamwork (*M_control_*=2.00, SD=1.00; *M_Intervention_*=2.25, SD=0.96),* t*(5)=-0.34, p=0.75. In the intervention group, the facilitators also reported that they had enough time to debrief (*MI_ntervention_*=2.50 *SD_Intervention_*=0. 587), whereas in the control group they were undecided (*M_control_*=0.33, *SD_control_*=2.08), *t*(2.2)=-1.75, p=.21. In the intervention condition, the facilitators described the TeamTAG as easy to handle (*M*=2.00, SD=1.16) and clearly structured (*M*=2.25, *SD*=0.96) and stated that it had helped them to conduct the debriefing (*M*=2.50, *SD*=0.58).

#### 3.5. Satisfaction with debriefings

Participants in both conditions expressed high levels of satisfaction with the debriefings across all stations (*M*=2.37-2.85; all *p*≥.06, see attachment 2 , here table 1).

#### 3.6. Importance of CRM principles

In the final survey, participants in both conditions rated all CRM principles as relevant or very relevant (*M*=1.94-2.77; all *p*≥.06, see attachment 2 , here table 2).

#### 3.7. Effect on teamwork

To test whether the type of debriefing would have a direct effect on teamwork, an ANCOVA was conducted with the TEAM sum score of the last (6^th^) case as the dependent variable, the condition (type of debriefing) as the independent variable, and the following covariates: CRM baseline, TEAM sum score of the 1^st^ case, and type of the 6^th^ case. The analysis revealed no effect of the type of debriefing on teamwork in the last case (*F*(1,1)=7.38, *p*=.23).

## 4. Discussion

The present study compares the effects of debriefings after simulated emergency situations with and without the use of a guideline called TeamTAG [28]. 

Our analyses show that by using the guideline, the repetition of debriefing topics is avoided, new topics are addressed, and the CRM principles that are rated as relevant for the specific simulation are actually addressed. Thus, if certain learning objectives are to be achieved, the use of a structuring cognitive aid can be helpful. For example, this approach can be used when teaching the basics of teamwork. Our observation that the topics in the control condition were more heterogeneous, and that the number of repetitions was higher, can be seen as signs of a stronger focus on the learners’ needs. Thus, debriefing without a guideline appears to be more learner-focused. Alternatively, it is possible that the repetitions are caused by a lack of new topics or ideas from participants and facilitators. Therefore, this approach seems to be more suitable for more experienced participants and facilitators to enable a differentiated debriefing [27], [38], [39].

Participants and the facilitators were satisfied with both debriefing methods. Only the time available for debriefing was not sufficient for some facilitators in the control group – a sign that the use of the guideline simplifies time management. This finding is a further argument for the use of a structuring cognitive aid in the case of inexperienced instructors or for debriefings in everyday clinical practice, for which usually little time is available [40].

The use of the guideline did not lead to the CRM principles being rated as more relevant than without the use of the guide. A possible explanation could be that the experience of teamwork during the simulations (which did not differ between conditions), and not so much the debriefing itself, influenced the perceived relevance of the CRM principles.

Finally, the present study could not detect any difference in the quality of teamwork depending on the debriefing. An expected improvement through greater pre-structuring could not be shown. Looking at this finding from another perspective, it means that the type of debriefing can be adapted to the learning objectives and learners without having to risk reductions in learner satisfaction or negative effects on trainees’ perceptions of CRM principles and teamwork quality. 

In this context, it must be pointed out that the simulated night shift focused on other aspects besides practising teamwork as well, such as experiencing the impact of fatigue on one’s ability to work. It is possible that this led to a lower cognitive capacity during the debriefings, which could also serve as an explanation for the lack of improvement in performance. In addition, for educational reasons, the roles of team leaders, members, and observers changed from case to case. This makes continuous improvement in teamwork quality more difficult and limits the generalisability of the study. In a study by Cheng et al. [27], which was able to show a better learning outcome after structured debriefings, the teams worked on virtually the same scenario before and after debriefing (with different starting points), whereas in the present study all six cases were different. Furthermore, a post-measurement of the teamwork quality some weeks or months after the SBT would be necessary to be able to make statements about possible long-term learning effects. After all, it has already been shown that participants in SBTs who planned to make changes after the training also implemented these changes in the majority of cases [41].

For a further investigation of the effects of TeamTAG in comparison with other debriefing scripts and guidelines, studies with fixed team structures and a larger number of groups should be conducted. Especially, the three teams of the control condition showed very heterogeneous results – for example, with regard to the number of topics discussed – which made it difficult to compare the findings to those from the intervention condition. These differences between the teams in the control and intervention conditions also suggest that, despite the structure applied by means of the TeamTAG cognitive aid, debriefing and its effects on learning remain highly dependent on the instructor [19], [27].

## 5. Conclusions

Cognitive aids such as TeamTAG can be used to predefine thematic focuses and the structure of debriefings and thus help to adapt them to the different levels of learners. Furthermore, the use of a guideline supports time management during debriefings.

## Data

Data for this article are available form the Dryad Digital Repository: https://doi.org/10.5061/dryad.02v6wwq2t [42]

## Funding

No specific funding was available for this study. JF was partially funded by the Quality Pact for Teaching from the German Federal Ministry of Education and Research (BMBF) (grant number: 01PL16036). WEH received research funding from the Swiss National Science Foundation and Mundipharma Research UK, as well as honoraria for consulting services from the AO Foundation Zurich. In each case, there was no connection to the present study. JEK received a Marie Sklodowska-Curie funding through Horizon 2020, an EU Framework Program for Research and Innovation (grant no. 894536, project “TeamUp”).

## Acknowledgements

We would like to thank everyone who participated in the study. Furthermore, we thank Prof. Dr. Stefan Schauber for his advice on the statistical analysis.

## Competing interests

The authors declare that they have no competing interests. 

## Supplementary Material

TeamTAG

Tables

## Figures and Tables

**Table 1:**
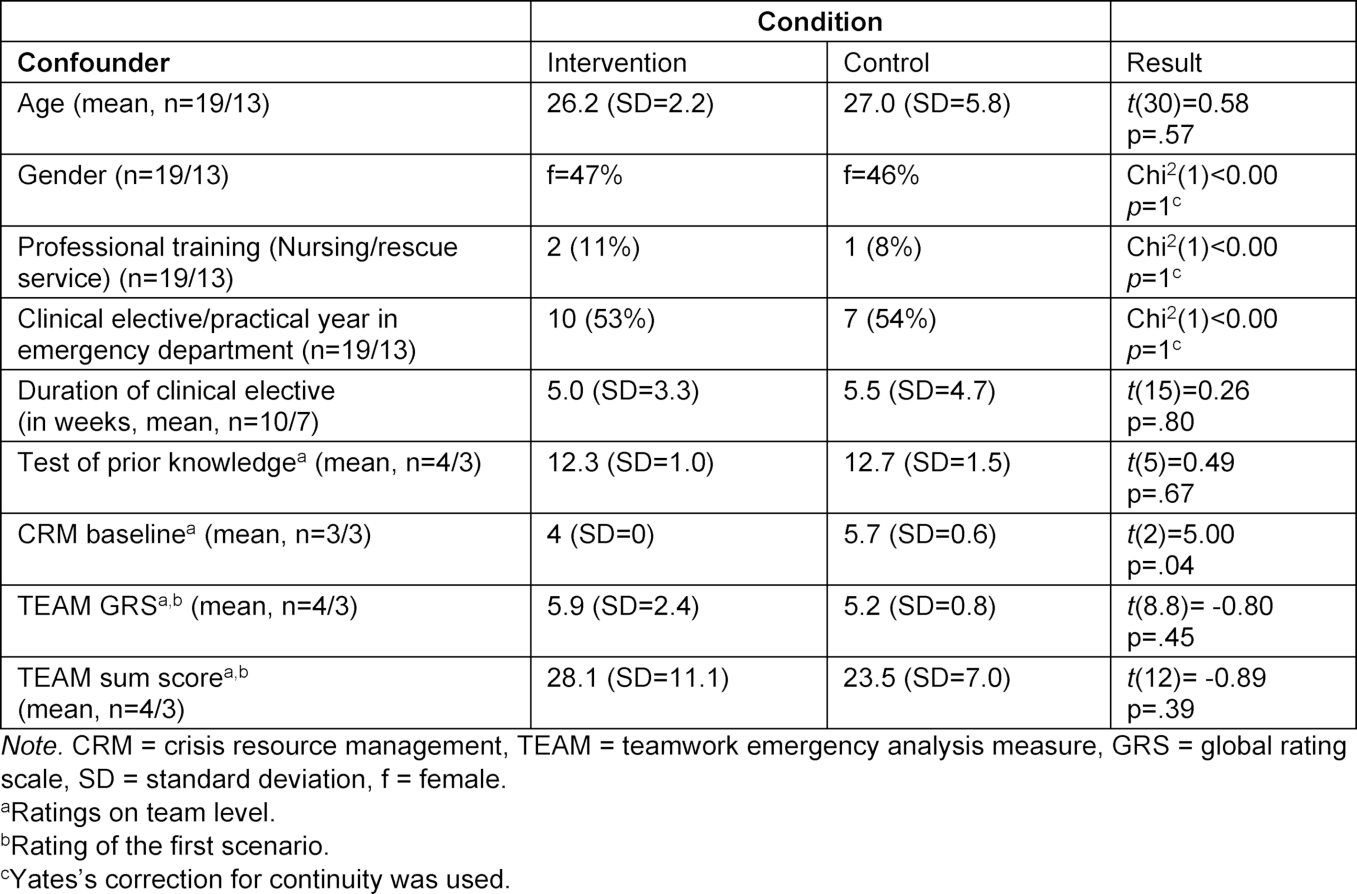
Baseline analysis
