# Supplementary material for: Effects of using a cognitive aid on content and feasibility of debriefings of simulated emergencies
Source: GMS J Med Educ. 2021 Jun 15;38(5):Doc95. doi: 10.3205/zma001491 (PMC8256120; doi:10.3205/zma001491)
Supplement: TeamTAG [file JME-38-5-95-s-001.pdf]

Attachment 1: TeamTAG

Key principle	Behavioural Marker	☒	Notes
Anticipate & Plan ahead	- agree on a plan with all team members<br>- think ahead and plan for all contingencies<br>- prepare a Plan B	☐<br>☐	
Set priorities dynamically	- identify and set priorities at the beginning<br>- pay attention towards changes which might become necessary / do not hold on to outdated concepts	☐<br>☐<br>☐	
Call for help early	- be aware of your own limits & the limits of your team<br>- set predefined criteria for asking for help<br>- know who and how you can call for help	☐<br>☐<br>☐	
Exercise leadership and followership	- as team leader:<br>• allocate team roles & tasks<br>• monitor progress<br>• pay attention to team members<br>• collect all information & make sure everyone is on the same page<br>- as a team member:<br>• be present and alert<br>• share your thoughts/doubts<br>• show appropriate self-care behaviour	 ☐<br>☐<br>☐<br>☐<br><br>☐<br>☐<br>☐	
Communicate effectively	- clear, assertive<br>- use Closed-Loop-Communication<br>- team leader receives all information	☐<br>☐<br>☐	
Re-evaluate repeatedly	- review the plan regularly, if / how it works<br>- respond to new information / arising problems etc.	☐<br>☐	

Briefing for instructors: Use TeamTAG during the simulation training to note the described behaviours. The blank space serves to note e.g. the specific situations, where a behaviour was shown or to note a quote. For the debriefing, one or two CRM principles should be selected and discussed in detail.

Attachment 1 to: Freytag J, Stroben F, Hautz WE, Penders D, Kämmer JE. *Effects of using a cognitive aid on content and feasibility of debriefings of simulated emergencies*. GMS J Med Educ. 2021;38(5):Doc95. DOI: 10.3205/zma001491
