# Supplementary material for: Effects of using a cognitive aid on content and feasibility of debriefings of simulated emergencies
Source: GMS J Med Educ. 2021 Jun 15;38(5):Doc95. doi: 10.3205/zma001491 (PMC8256120; doi:10.3205/zma001491)
Supplement: Tables [file JME-38-5-95-s-002.pdf]

Attachment 2

Table 1: Comparison of participants' satisfaction with debriefings in intervention and control groups

Case	Intervention (M, SD)	Control (M, SD)	*t*	*df*	*p*
Cardiology	2.58, 0.69	2.77, 0.44	0.88	30	.39
Neurology	2.74, 0.56	2.46, 0.88	-1.09	30	.20
Pulmonology	2.37, 1.12	2.54, 0.88	0.46	30	.65
Surgery	2.57, 0.51	2.67, 0.50	0.49	21	.67
Traumatology	2.47, 0.70	2.85, 0.38	1.95	28.76[a]	.06
Resuscitation	2.82, 0.41	2.71, 0.49	-0.49	16	.63

[a]Welch's correction was used

Table 2: Comparison of participants' ratings of the importance of CRM principles in intervention and control groups (final survey)

CRM principle	Intervention (M, SD)	Control (M, SD)	*t*	*df*	*p*
1	2.74, 0.45	2.77, 0.44	0.20	30	.84
2	2.42, 0.61	2.69, 0.48	1.35	30	.19
3	2.63, 0.60	2.62, 0.51	-0.08	30	.94
4	2.56, 0.62	2.38, 0.51	-0.82	29	.42
5	2.53, 0.51	2.62, 0.51	0.49	30	.63
6	2.42, 0.84	2.69, 0.48	1.05	30	.30
7	2.68, 0.48	2.62, 0.65	-0.35	30	.73
8	2.42, 0.61	2.38, 0.77	-0.15	30	.88
9	2.53, 0.70	2.23, 1.01	-0.98	30	.34
10	2.68, 0.58	2.00, 1.47	-1.59	14.60[a]	.13
11	2.32, 0.67	2.75, 0.45	1.97	29	.06
12	2.58, 0.51	2.77, 0.44	1.13	28.25[a]	.27
13	2.61, 0.61	2.54, 0.66	-0.32	20	.75
14	1.94, 1.21	2.62, 0.65	1.81	29	.08
15	2.32, 0.82	2.54, 0.66	0.81	30	.42

[a]Welch's correction was used
